# A Case of Severe Neonatal Neutropenia Secondary to Late-Onset Sepsis From E. coli Omphalitis

**DOI:** 10.7759/cureus.101601

**Published:** 2026-01-15

**Authors:** Hadi Fakih, Hussein Hussein, Fatima Fakih

**Affiliations:** 1 Pediatrics, Sheikh Ragheb Harb University Hospital, Nabatieh, LBN; 2 Pediatrics, Lebanese University Faculty of Medical Sciences, Beirut, LBN; 3 Neonatology, Lebanese University Faculty of Medicine, Beirut, LBN

**Keywords:** e. coli, granulocyte colony-stimulating factor, late-onset neonatal sepsis, neonatal neutropenia, omphalitis, whole exome sequencing

## Abstract

Severe neonatal neutropenia, defined as an absolute neutrophil count (ANC) of <500/µL, is a critical hematologic finding that signals either a severe consumptive process or a primary bone marrow disorder. We present the case of an eight-day-old, full-term female infant who presented with fever and irritability, later diagnosed with severe neutropenia (ANC: 400/µL) and elevated C-reactive protein (CRP) (150 mg/L). Omphalitis was identified as the source, with pus culture growing pan-sensitive *Escherichia coli*, confirming late-onset neonatal sepsis (LONS). The patient was managed with targeted intravenous ceftazidime and a short course of granulocyte colony-stimulating factor (G-CSF). A comprehensive workup, including a bone marrow aspirate and whole exome sequencing (WES), was performed to rule out congenital etiologies; WES returned negative, supporting the diagnosis of acquired, sepsis-induced neutropenia. On follow-up at two months of age, the infant was asymptomatic and thriving well and had a normal complete blood count with differential (CBCD). This case underscores the importance of a systematic diagnostic approach to neonatal neutropenia, highlighting the differential diagnosis and the evolving role of advanced genetic testing such as WES in providing definitive diagnostic and prognostic clarity, thereby guiding appropriate management.

## Introduction

Neonatal neutropenia is a common but clinically significant finding, affecting up to 38% of very-low-birth-weight infants [1]. It is defined as an absolute neutrophil count (ANC) below the fifth percentile for postnatal age, with severe neutropenia constituting an ANC of <500/µL, a threshold associated with a markedly increased risk of invasive bacterial and fungal infections [2]. The etiologic spectrum is broad, ranging from benign, transient conditions such as idiopathic neutropenia of prematurity to life-threatening causes such as severe sepsis, alloimmune disorders, and congenital bone marrow failure syndromes (e.g., Kostmann syndrome and Shwachman-Diamond syndrome) [3].

Late-onset neonatal sepsis (LONS), occurring after 72 hours of life, remains a leading cause of neonatal morbidity. While pathogens such as group B *Streptococcus* and *Escherichia coli* are common, omphalitis, defined as an infection of the umbilical stump, is a recognized but less frequent portal of entry in the modern era [4]. The diagnostic challenge lies in differentiating sepsis-induced consumptive neutropenia from primary hematologic disorders, a distinction with profound implications for acute management and long-term prognosis.

We report a case of an otherwise healthy term neonate who developed severe neutropenia secondary to culture-proven *E. coli* LONS originating from omphalitis. This report details the comprehensive diagnostic workup, including the use of whole exome sequencing (WES), and discusses the critical differential diagnosis and management principles for neonatal neutropenia.

## Case presentation

An eight-day-old, full-term (38 5/7 weeks) female infant presented to the neonatal intensive care unit (NICU) with a history of two episodes of fever (38.6°C rectally) over six hours, accompanied by new-onset irritability. She was born via elective repeat cesarean section to a 35-year-old G4P2A1L1 mother. The prenatal course was complicated by a maternal urinary tract infection at eight months of gestation; group B *Streptococcus* screening was not performed. Delivery was uncomplicated with Apgar scores of 9 and 10 at one and five minutes, respectively. She was discharged home on exclusive breastfeeding.

Family history was noncontributory, with no known consanguinity between parents and no prior history of neonatal neutropenia, recurrent severe infections, or primary immunodeficiency disorders in the immediate or extended family. Maternal blood investigations, including a complete blood count with differential (CBCD), were within normal limits. Both direct and indirect Coombs tests performed on the neonate were negative, ruling out hemolytic disease as a confounding factor.

Two days prior to admission (day of life {DOL} 6), she was seen in a private clinic for umbilical erythema without systemic symptoms and was prescribed topical antibiotic ointment. On the day of admission (DOL 8), she developed high-grade fever (38.6°C) and irritability.

**Figure 1:**
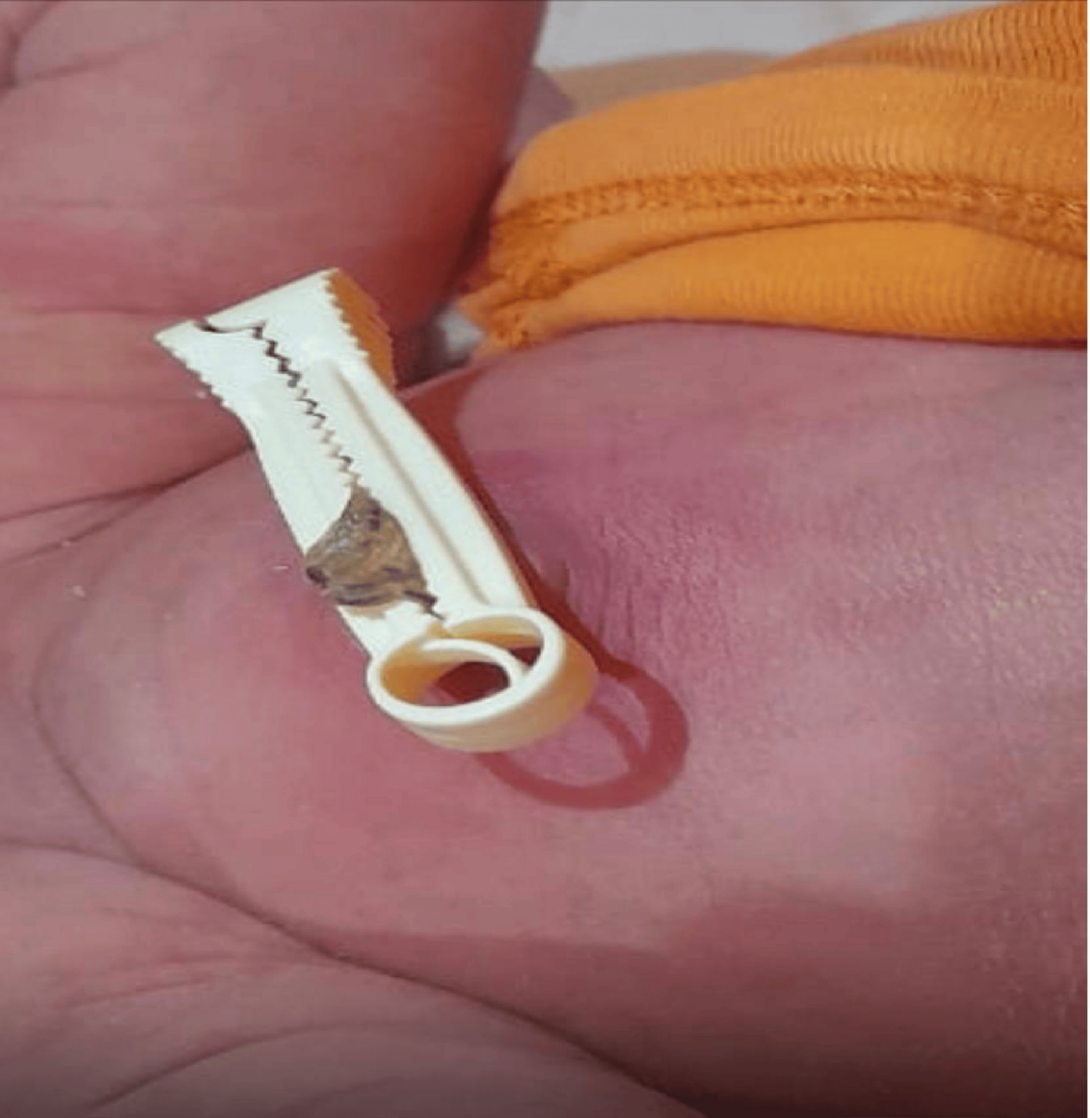
Significant periumbilical erythema and inflammation consistent with omphalitis on admission.

On admission, the infant was febrile, irritable, and plethoric. Vital signs were stable. Physical examination was significant for the findings shown in Figure 1: an inflamed umbilical stump with surrounding cellulitis. The anterior fontanelle was soft and flat. Cardiorespiratory, abdominal, and neurological examinations were otherwise normal.

Initial laboratory evaluation revealed profound neutropenia and systemic inflammation (Table 1). A peripheral blood smear showed activated lymphocytes without immature myeloid forms or dysplastic features. Blood and cerebrospinal fluid cultures were subsequently negative, likely due to antecedent antibiotic exposure. However, a pus swab from the umbilical site grew *Escherichia coli*, pan-sensitive to antibiotics.

**Table 1:** Initial laboratory findings on admission.

Tests	Patient Results	Normal Reference for Age
Absolute Neutrophil Count (ANC)	400/µL	2700-13000/µL [5]
C-reactive Protein (CRP)	150 mg/L	<10 mg/L
White Blood Cell (WBC) Count	2.1 × 10³/µL	5.0-21.0 × 10³/µL
Platelets	210 × 10³/µL	150-450 × 10³/µL

Empiric therapy for LONS was initiated with intravenous ampicillin and cefotaxime. On hospital day 3, following culture results, therapy was de-escalated to intravenous ceftazidime to target the *E. coli* and mitigate the risk of antibiotic-associated neutropenia. Given the severity and persistence of neutropenia, adjuvant therapy with subcutaneous granulocyte colony-stimulating factor (G-CSF) (10 mcg/kg/day) was administered for three days. The serial hematologic response is detailed in Table 2.

**Table 2:** Serial hematologic trends during hospitalization. DOL, day of life; WBC, white blood cell; ANC, absolute neutrophil count; G-CSF, granulocyte colony-stimulating factor

Hospital Day (DOL)	WBC (×10³/µL)	ANC (/µL)	Clinical Event/Intervention
1 (8)	2.1	400	Admission and Start of Empiric Antibiotics
2 (9)	2.5	450	-
3 (10)	3.8	1200	Switch to Ceftazidime and Start of G-CSF
4 (11)	8.2	4200	G-CSF Day 2
5 (12)	10.5	5800	G-CSF Day 3 and ANC Recovery
7 (14)	9.1	5100	Antibiotics Continued
10 (17)	8.7	4900	Discharge

A comprehensive diagnostic workup for persistent severe neutropenia was undertaken. A toxoplasmosis, other agents, rubella, cytomegalovirus, and herpes simplex (TORCH) screen and a multiplex polymerase chain reaction (PCR) for neonatal pathogens (Neuro 9 panel) were negative. Peripheral blood flow cytometry showed no evidence of a lymphoproliferative disorder. A bone marrow aspirate revealed a normocellular marrow with a left-shifted myeloid series and a severely depleted mature neutrophil storage pool, consistent with peripheral consumption. Whole exome sequencing (WES) was sent to definitively rule out congenital neutropenia syndromes and returned negative for pathogenic variants in genes associated with severe congenital neutropenia (e.g., *ELANE*, *HAX1*, *SBDS*, and *G6PC3*) and other primary immunodeficiencies.

The patient showed rapid clinical improvement. She completed a 10-day course of ceftazidime, was weaned off all support, and was discharged home in stable condition on exclusive breastfeeding. On follow-up at two months of age, the infant was completely asymptomatic, thriving well with appropriate weight gain, and a repeat complete blood count revealed a normal white blood cell count and ANC, confirming the transient and resolved nature of the neutropenia.

## Discussion

This case exemplifies the classic presentation of severe, acquired neonatal neutropenia driven by bacterial sepsis. The pathophysiology is primarily consumptive: neutrophils are sequestered at the site of infection (omphalitis) and within the reticuloendothelial system, depleting the circulating pool faster than bone marrow production can compensate [6]. The bone marrow findings of a depleted storage pool with an active proliferative pool are hallmark features of this process.

The principal diagnostic imperative in a neonate with severe neutropenia is to distinguish between increased peripheral destruction/consumption (e.g., sepsis and alloimmune neutropenia) and decreased bone marrow production (e.g., congenital syndromes and marrow infiltration). Our diagnostic approach followed a logical sequence beginning with initial triage via a complete blood count with differential, inflammatory markers (C-reactive protein {CRP}), and comprehensive cultures. This was followed by etiologic delineation using tools such as the immature-to-total neutrophil ratio, maternal antibody screening, and bone marrow examination. The final, definitive step involved the exclusion of congenital causes through advanced genetic testing with whole exome sequencing (WES).

The differential diagnosis for neonatal neutropenia is broad. Alloimmune neonatal neutropenia, analogous to the hemolytic disease of the newborn, typically presents in an otherwise well infant with isolated neutropenia that resolves as maternal antibodies clear. Congenital neutropenia syndromes (e.g., Kostmann syndrome), in contrast, present with profound, persistent neutropenia from birth and a history of recurrent, severe infections often in the absence of a clear septic focus; they carry a lifelong risk of infection and potential for malignant transformation [3].

The role of whole exome sequencing (WES) in this context is of paramount diagnostic and prognostic value. For cases of severe, persistent, or recurrent neutropenia where acquired causes are not definitive, WES serves as a powerful tool to identify mutations in over 30 genes associated with congenital neutropenia and related immunodeficiencies [7]. A positive WES result provides a definitive diagnosis, informs genetic counseling, predicts clinical course (e.g., need for lifelong G-CSF and the risk of leukemia), and can guide family planning. Conversely, a negative WES result, as in our patient, is equally valuable. It effectively rules out known genetic etiologies; strongly supports an acquired, reactive cause (such as sepsis); and predicts an excellent long-term hematologic prognosis with a low risk of recurrence. This genetic certainty prevents unnecessary long-term G-CSF therapy and alleviates significant parental anxiety.

The management of sepsis-induced neutropenia is twofold: source control with appropriate antimicrobials and the supportive management of the cytopenia. The use of G-CSF in neonatal sepsis remains adjunctive and controversial, not a standard of care [8]. It may be considered in cases of profound neutropenia (ANC: <500/µL) with clinical deterioration or evidence of impaired marrow reserve, as it was here, to shorten the duration of neutropenia and potentially mitigate infection risk.

## Conclusions

This case demonstrates that severe neonatal neutropenia is a critical but often transient complication of late-onset sepsis, as confirmed in our patient with *E. coli* omphalitis. A systematic diagnostic approach, including detailed history, laboratory evaluation, bone marrow examination, and advanced genetic testing via whole exome sequencing (WES), is essential to exclude primary hematologic disorders. In this instance, the negative WES and the infant's subsequent asymptomatic recovery with normal hematologic parameters at two-month follow-up definitively supported an acquired, sepsis-induced etiology. This underscores the importance of targeted antimicrobial therapy, with the consideration of adjunctive G-CSF in profound cases, and highlights the prognostic value of comprehensive genetic workup in guiding management and reassuring prognosis.

## References

[1] Christensen RD, Henry E, Wiedmeier SE, Stoddard RA, Lambert DK (2006). Low blood neutrophil concentrations among extremely low birth weight neonates: data from a multihospital health-care system. J Perinatol.

[2] Maheshwari A (2014). Neutropenia in the newborn. Curr Opin Hematol.

[3] Donadieu J, Fenneteau O, Beaupain B, Mahlaoui N, Chantelot CB (2011). Congenital neutropenia: diagnosis, molecular bases and patient management. Orphanet J Rare Dis.

[4] Mullany LC, Darmstadt GL, Khatry SK, LeClerq SC, Katz J, Tielsch JM (2006). Impact of umbilical cord cleansing with 4.0% chlorhexidine on time to cord separation among newborns in southern Nepal: a cluster-randomized, community-based trial. Pediatrics.

[5] Manroe BL, Weinberg AG, Rosenfeld CR, Browne R (1979). The neonatal blood count in health and disease.I. Reference values for neutrophilic cells. J Pediatr.

[6] Machimoto N, Baba Y, Takaoka Y, Shoji H, Shimizu T (2023). A case of omphalitis revealing alloimmune neonatal neutropenia. Cureus.

[7] Arikoglu T, Kuyucu N, Germeshausen M, Kuyucu S (2015). A novel G6PC3 gene mutation in severe congenital neutropenia: pancytopenia and variable bone marrow phenotype can also be part of this syndrome. Eur J Haematol.

[8] Carr R, Modi N, Doré C (2003). G-CSF and GM-CSF for treating or preventing neonatal infections. Cochrane Database Syst Rev.

